# Accreditation Council for Graduate Medical Education Milestones for Emergency Medicine Residency Training Incorporated into First- and Second-Year Medical Student Elective

**DOI:** 10.30476/jamp.2021.88982.1360

**Published:** 2021-07

**Authors:** CHRISTINA Y. CANTWELL, JONATHAN B. LEE, SOHEIL SAADAT, NICHOLAS BOVE, SANGEETA SAKARIA, WARREN WIECHMANN, ALISA WRAY, SHANNON TOOHEY

**Affiliations:** 1 University of California Irvine Medical Center, Department of Emergency Medicine, 101 The City Drive South, Orange, CA 92868, USA

**Keywords:** Curriculum, Medical education, Graduate medical education, Emergency medicine

## Abstract

**Introduction:**

As part of its Next Accreditation System, the Accreditation Council for Graduate Medical Education and the American Board of Emergency Medicine describe 6 competencies containing 23 sub-competencies graded by milestones ranging from level 1 (expected of an incoming intern) to level 5 (demonstrates abilities of an attending) that are used to track resident training progression. To the best of our knowledge, there have been no studies introducing a milestones-based curriculum to medical students prior to their introduction to the wards, so we sought to determine the effects that a pre-clinical Emergency Medicine Interest Group (EMIG) Milestones Elective would have on preparing the students interested in Emergency Medicine (EM) as a specialty to meet the level 1 milestones prior to their intern year.

**Methods:**

The elective hosted 15 events throughout the academic year, and pre- and post-curriculum surveys were administered. Thirteen first- and second-year medical students at our institution who completed the elective self-reported their perception of preparedness for each level 1 milestone in the 19 sub-competencies. A repeated measures design was used through identical pre- and post-curriculum surveys to determine any changes in self-reported preparedness for meeting level 1 milestones after completing the elective using Wilcoxon Signed Ranks Test.

**Results:**

There was a significant increase in the median scoring from 1 to 2 (p=0.027) in overall self-reported preparedness for meeting the level 1 milestones included in the elective, as well as significant increases in subcategories across competencies 1-4 outlined by the ACGME. There was no significant increase in preparedness for professionalism or interpersonal communication competencies. There was no significant increase in interest in EM as a result of the elective.

**Conclusion:**

Implementing a milestones-based curriculum during the pre-clinical years shows improved self-reported preparedness of students interested in pursuing EM for meeting level 1 milestones prior to residency. Additionally, a specialty-based elective such as this one offered through EMIG may further increase interest in the field during pre-clinical years.

## Introduction

In 2013, the Accreditation Council for Graduate Medical Education (ACGME) implemented its Next Accreditation System (NAS) that requires semiannual evaluation of the milestones that are expected of residents throughout their training ( 1). This move was due in part to shifting attitudes regarding medical education, specifically moving towards an outcome-driven system of evaluating success ( 2). For Emergency Medicine (EM), the American Board of Emergency Medicine describes 23 sub-competencies with milestones that range from level 1 (expected of an incoming resident/intern) to level 5 (exceptional residents that demonstrate abilities of an attending). 

Since implementation of NAS, studies of incoming interns have found that many fall short of meeting level 1 milestones ( 3- 4). Santen et al. previously surveyed EM interns across the United States and reported up to 39% of interns reported never receiving instruction on certain milestones ( 3). Additionally, a previous observational study on incoming EM interns found a wide variability ranging from 48-93% competency in the milestones assessed ( 4). Challenges in implementing competency-based medical education such as NAS include barriers to creating curricula that individualize learning plans and inconsistent assessment of milestones ( 5). To our knowledge, there have not yet been studies on introducing a milestones-based curriculum in the pre-clinical years, typically the first and second year of traditional medical school curricula. Here, we describe a curriculum developed for medical students to introduce milestones prior to entering the residency stage. 

This study took place at a medical school with a robust ultrasound curriculum that showed success in early integration and longitudinal development of ultrasound skills throughout medical school ( 7- 8). We sought to determine the effects that a pre-clinical Emergency Medicine Interest Group (EMIG) Milestones Elective would have on preparing students interested in EM as a specialty to meet the level 1 milestones prior to graduating medical school.

The EMIG Milestones Elective’s objective was to prepare the students who complete the elective to meet 19 of the 23 level 1 milestones (4 were omitted, as they are better suited for MS3 and MS4 years in a clinical setting). As a result of the study, we sought to determine the effect, if any, that the elective had on 1) preparing students to meet milestone expectations, and 2) impacting the level of interest expressed by the student in pursuing Emergency Medicine as a specialty. Additionally, the results of the survey would allow us to identify parts of the curriculum to improve for future years. Here, we report significant increases in self-reported preparedness for meeting the majority of the level 1 milestones included in the study. 

## Methods

This study was reviewed by the Institutional Review Board and classified as exempt with a waived requirement for signed informed consent. A Study Information Sheet was provided to students via email and on the first page of the electronic survey with response buttons to indicate consent. Students were allowed to participate in the elective regardless of participation in our study without penalty. 

There were 23 sub-competencies outlined by the ACGME. Four of the 23 were omitted in designing the curriculum because they were better suited for third- and fourth-year training. The four omitted sub-competencies were PC8 multi-tasking, SBP1 patient safety, SBP3 technology, and PROF2 accountability as these are better taught in a clinical setting during the third- and fourth-year medical school training. The decision to omit these sub-competencies was made by the elective coordinator with guidance from the faculty advisor who was also associate residency program director at the time and well-versed in milestone requirements and residency education. The remaining 19 sub-competencies were more broadly categorized into 6 competencies based on ACGME guidelines: patient care, medical knowledge, system-based practice, practice-based learning and improvement, professionalism, and interpersonal and communication skills. The 19 level 1 milestones are outlined in Table 1. Data regarding competencies 5 and 6 were combined in our analysis due to the similarity in competency features. 

**Table1:** Categorization of ACGME milestones included in the EMIG Milestones Elective a indicates level 1 milestone not included in the elective

Competency	Sub-competency	Level 1 Milestone
1: Patient Care	PC1: Emergency stabilization	Recognizes abnormal vital signs.
	PC2: Performance of focused H&P	Performs and communicates a reliable, comprehensive history and physical exam.
	PC3: Diagnostic studies	Determines the necessity of diagnostic studies.
	PC4: Diagnosis	Constructs a list of potential diagnoses based on chief complaint and initial assessment.
	PC5: Pharmacotherapy	Knows the different classifications of pharmacologic agents and their mechanism of action. Consistently asks patients for drug allergies.
	PC6: Observation and reassessment	Recognizes the need for patient re-evaluation.
	PC7: Disposition	Describes basic resources available for care of the emergency department patient.
	PC8: Multi-tasking	Manages a single patient amidst distractionsa
	PC9: General approach to procedures	Identifies pertinent anatomy and physiology for a specific procedure.
		Uses appropriate Universal Precautions.
	PC10: Airway management	Describes upper airway anatomy.
		Performs basic airway maneuvers or adjuncts (jaw thrust/chin lift/oral airway/nasopharyngeal airway) and ventilates/oxygenates patient using BVM.
	PC11: Anesthesia and acute pain management	Discusses with the patient indications, contraindications and possible complications of local anesthesia.
		Performs local anesthesia using appropriate doses of local anesthetic and appropriate technique to provide skin to sub-dermal anesthesia for procedures.
	PC12: Other diagnostic and therapeutic procedures: Goal-directed Focused Ultrasound	Describes the indications for emergency ultrasound.
	PC13: Other diagnostics and therapeutic procedures: Wound management	Prepares a simple wound for suturing (identify appropriate suture material, anesthetize wound and irrigate).
		Demonstrates sterile technique Places a simple interrupted suture.
	PC14: Other diagnostics and therapeutic procedures: Vascular access	Performs a venipuncture.
		Places a peripheral intravenous line Performs an arterial puncture.
2: Medical Knowledge	MK: Medical knowledge	Passes initial national licensing examinations (e.g., USMLE Step 1 and Step 2 or COMLEX Level 1 and Level 2).
3: System Based Practice	SBP1: Patient safety	Adheres to standards for maintenance of a safe working environment Describes medical errors and adverse eventsa.
	SBP2: Systems based management	Describes members of ED team (e.g., nurses, technicians, and security).
	SBP3: Technology	Uses the Electronic Health Record (EHR) to order tests, medications and document notes, and respond to alerts Reviews medications for patientsa.
4: Practice Based Learning and Improvement	PBLI: Practice-based performance improvement	Describes basic principles of evidence-based medicine.
5: Professionalism	PROF1: Professional values	Demonstrates behavior that conveys caring, honesty, genuine interest and tolerance when interacting with a diverse population of patients and families.
	PROF2: Accountability	Demonstrates basic professional responsibilities such as timely reporting for duty, appropriate dress/grooming, rested and ready to work, delivery of patient care as a functional physician.
		Maintains patient confidentially.
		Uses social media ethically and responsibly Adheres to professional responsibilities, such as conference attendance, timely chart completion, duty hour reporting, and procedure reportinga.
6: Interpersonal and Communication Skills	ICS1: Patient centered communication	Establishes rapport with and demonstrate empathy toward patients and their families.
		Listens effectively to patients and their families.
	ICS2: Team management	Participates as a member of a patient care team.

EMIG: Emergency Medicine Interest Group; ACGME: Accreditation Council for Graduate Medical Education

Convenience sampling was used to gather our data. Participants were first- and second-year medical students enrolled in the milestones elective. A survey assessing the level of interest in EM, overall preparedness for an intern year in EM, and readiness for meeting the Level 1 skills for each of the 19 included milestones was designed in Qualtrics Survey Software. The responses were scored based on a Likert scale (i.e. not prepared at all, somewhat prepared, neutral, very prepared, and extremely prepared). A repeated measures design was used, in which the same variables were measured on the same sample before and after the curriculum. The survey was first administered at the beginning of the academic year prior to any elective events (pre-curriculum survey). Respondents taking the pre-curriculum survey were de-identified and assigned an anonymous, random 6-digit identifier used to track the survey results at the end of the year (post-curriculum survey). The post-curriculum survey contained the same questions and answer choices as the pre-curriculum survey. Due to the novelty and specific focus of our study design, the questions used in our survey have not been validated.  

There were 15 events throughout the year: Wilderness Medicine, Intro to EM Talk, Procedures Workshop, five Talk Shops with EM attendings, Research Opportunities dinner, Shadowing, Jeopardy, Matching into EM Panel, Disaster Medicine, Cadaver Workshop, and Post-Match Panel. Sub-competencies were assigned to events based on the event type (Table 2). These assignments were also discussed with the faculty member overseeing the elective. This advisor was also well-versed in what each event entailed. Students earned credit for sub-competencies assigned to a particular event by attending. Credit for the elective was earned by attending the combination of events to satisfy all 19 sub-competencies and at least eight events. After all events were held, the post-curriculum survey was administered using the 6-digit identifier for longitudinal tracking. 

**Table 2:** Milestones assigned to each event and event descriptions

Event	Description	Sub-competencies
Wilderness Medicine	Camping weekend and educational conference in the San Bernardino Mountains instructed by EM physicians.	PC1, PC5, PC6, PC7, PC9, PC10, PC13, PC14, MK, ICS1, ICS2
Intro to EM Talk	EM attendings introduce the field and dynamic flow in the emergency department.	PC3, PC5, PC6, MK, PROF1, ICS1
Procedures Workshop	Four rotating stations of suturing, ultrasound-guided IV insertion, IV access, intubation.	PC1, PC2, PC3, PC4, PC5, PC6, PC7, PC9, PC10, PC11, PC12, PC13, PC14, PBLI
Talk Shops with EM Attendings	Five dinners held throughout the year at ED attendings’ houses.	PC7, SBP2, PROF1, ICS1, ICS2
Research Opportunities Dinner	Dinner with ED attendings where ongoing research projects are introduced.	MK, SBP2, PBLI
Shadowing	ED shadowing scheduled by students based on availability.	Varied. Students were allowed to choose up to 7 milestones per day of shadowing for credit with a brief description of cases seen that satisfy the milestones chosen.
Jeopardy	Test-your-knowledge of EM related topics.	PC1, PC2, PC3, PC4, PC5, PC6, PC7, PC10, PC11, PC12, PC13, PC14, MK, PBLI, ICS1, ICS2
Matching into EM Panel	Attendings describe the path to matching into EM.	SBP2, PROF1, ICS1, ICS2
Disaster Medicine Talk	Lunch talk with EM physician describing the role of disaster medicine.	PC1, PC4, PC9, PC13, ICS2
Cadaver Workshop	Procedures demonstrated on fresh tissue from cadaveric donors.	PC1, PC2, PC4, PC9, PC10, PC11, PC12, MK, PBLI
Post-Match Panel	Graduating MS4s discuss their path to matching into EM.	SBP2, PROF1

The elective was graded based on completion for transcript notation and without penalty if a student did not complete the elective. No letter grades were assigned, and participation in the survey was voluntary and anonymous. Forty-six first- and second-year medical students signed up for the elective, and 22 of the 46 completed the elective for credit. Twenty-two students agreed to participate in the study and took the pre-curriculum survey. Thirteen out of those 22 students who completed the initial survey also completed the post-curriculum survey. Students who did not complete the elective were not included in our study due to an incomplete data set. We used these 13 sets of data for our analysis. 

### Statistical analysis

The responses were re-coded into 1 (not prepared/interested at all) to 5 (extremely prepared/interested). Distribution of milestones are presented as median and interquartile range (quartile 1 to quartile 3). In case of competencies 1 and 6, the median and interquartile range (IQR) of all milestones that comprised those competencies were calculated. The statistical significance of any difference between pre- and post- curriculum was calculated by using Wilcoxon Signed Ranks Test. The Wilcoxon Signed Ranks Test was used because the survey design repeated measurements before and after the curriculum on the same sample, as the surveys administered asked identical questions to the same respondents. We used IBM SPSS statistics 26 for data analysis.

### Ethical Consideration

Some of the members involved in the creation of this study were faculty who worked directly with the study participants in other aspects of training. Participation in the study was optional and participants could withdraw at any time without penalty or academic repercussions. The data collected was not traceable to individual respondents due to the use of anonymous, randomly-assigned identifiers. The survey questions assessed self-identified level of interest/preparedness in the field of emergency medicine in general and not specific to our academic institution. There was no reference to our institution's faculty or affiliates, so participants could answer survey questions honestly without any impact on transcript grades.

## Results

**Figure 1:**
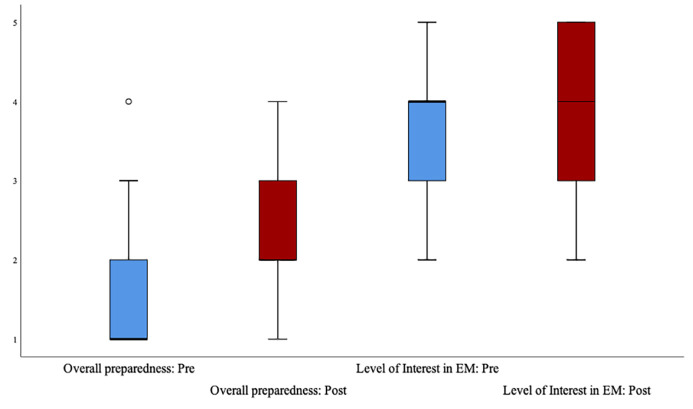
Self-reported level of preparedness and level of interest in emergency medicine (EM), before and after completing the EMIG Milestones Elective, EMIG : Emergency Medicine Interest Group, ACGME: Accreditation Council for Graduate Medical Education

There were 13 sets of data included for analysis. The median (IQR) for overall preparedness for an intern year in an EM residency was 1 (1 to 2) before the curriculum and 2 (2 to 3) after the curriculum (Figure 1, p=0.027). The median level of interest in EM was 4 (3 to 5) before the curriculum. The median level of interest in EM remained 4 (3 to 5) after the curriculum (Figure 1, p=0.317).

**Figure 2:**
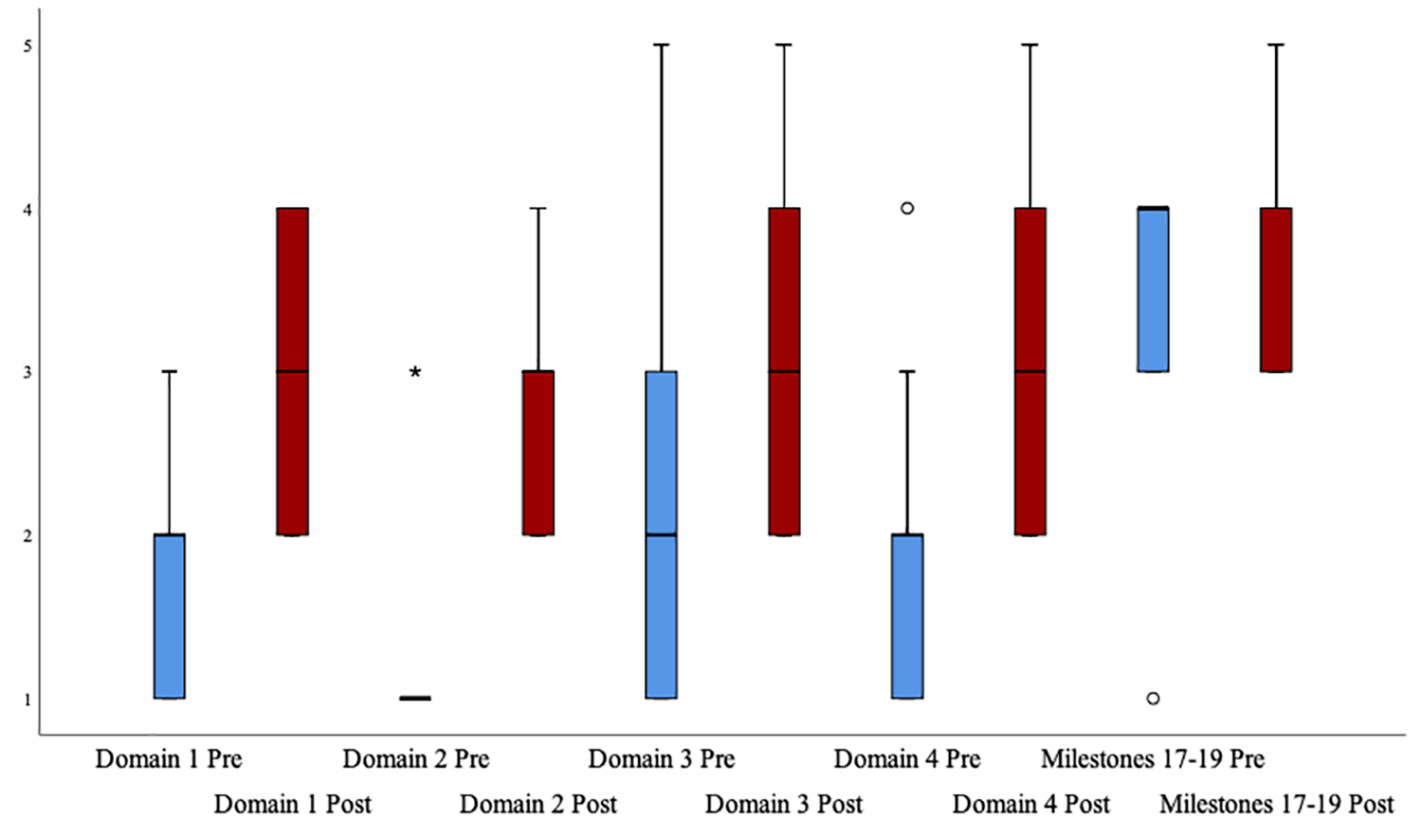
Level of readiness for level 1 milestones, before and after the EMIG Milestones Elective. Increased readiness was found in competencies 1, 2, 3, and 4.

In assessing perceived preparedness for each of the 19 level 1 milestones grouped by competency, there were significant increases in competencies 1-4 (Figure 2). The categories and their corresponding statistical p-values are displayed in Table 3. For competency 1 (patient care, sub-competencies PC1-PC14), the median score was 2 (1 to 3) prior to the elective and increased to 3 (2 to 4) after elective (p=0.004) (Table 3). 

**Table3:** Pre- and post-curriculum measures and the statistical significance (based on Wilcoxon Signed Ranks Test)

Measure	Intervention	N	Mean	Median	Min	Max	^1st^ quartile	^3rd^ quartile	Statistical test
									Z	P
Overall preparedness for intern year in EM residency	Pre	13	1.5	1	1	4	1	2	-2.209	0.027
	Post	13	2.4	2	1	4	2	3		
Level of Interest in EM	Pre	13	3.8	4	2	5	3	5	-1.000	0.317
	Post	13	4.1	4	2	5	3	5		
Competency 1 (Patient Care)	Pre	13	1.8	2	1	3	1	3	-2.889	0.004
	Post	13	2.9	3	2	4	2	4		
Competency 2 (Medical Knowledge)	Pre	13	1.3	1	1	3	1	1	-3.134	0.002
	Post	13	2.8	3	2	4	2	4		
Competency 3 (Systems-Based Management)	Pre	13	2.4	2	1	5	1	4	-2.081	0.037
	Post	13	3.1	3	2	5	2	4		
Competency 4 (Practice-Based Performance Improvement)	Pre	13	1.9	2	1	4	1	3	-2.889	0.004
	Post	13	3.0	3	2	5	2	4		
Competency 5 (Professionalism)	Pre	13	3.2	3	1	5	3	4	-1.867	0.062
	Post	13	3.8	4	3	5	3	5		
Competency 6 (Interpersonal Skills)	Pre	13	3.3	3	2	5	3	4	-1.308	0.191
	Post	13	3.6	4	3	5	3	4		
Competencies 5-6 (Professionalism and Interpersonal skills)	Pre	13	3.4	4	1	4	3	4	-1.190	0.234
	Post	13	3.7	4	3	5	3	4		

N: Count, Min: Minimum, Max: Maximum

The median score for preparedness in competency 2 (medical knowledge, MK) was 1 prior to the elective; median level of preparedness increased to 3 (2 to 4) after the elective (p=0.002) (Figure 2). Competency 3 (system-based practice, SBP2) showed a median increase from 2 to 3 (p=0.037), and competency 4 (practice-based learning and improvement, PBLI) also showed a median increase from 2 to 3, (p=0.004) (Figure 2). Competencies 5 and 6 (professionalism and interpersonal and communication skills, PROF1 and ICS1-ICS2) did not show a significant increase from the post-curriculum survey. For these milestones, the medians for pre- and post-curriculum were 4 (p=0.234) (Figure 2). 

## Discussion

Significant increases in the students’ perceived preparedness were seen in 16 out of the 19 level 1 milestones included in the elective as well as overall. The competency that showed the most significant increase was medical knowledge (sub-competency MK). Within the elective, PC1 and MK (medical knowledge) had the highest number of events that qualified for milestone credit, which may have played a proportional role in preparing students to feel they could meet the level 1 milestones for those sub-competencies. As an elective geared towards first- and second-year students, it is also expected that initial medical knowledge may naturally increase with more time and exposure to medicine, not only in the field of EM, but generalizable to the time spent in the medical school environment.  

The next competencies to show the highest significance in increased preparedness were patient care (sub-competencies PC1-PC14) and practice-based learning and improvement (sub-competency PBLI). Patient care encompasses the greatest number of sub-competencies, and 11 out of the 15 events covered at least one sub-competency in this broader competency. Therefore, an increased frequency of events that incorporate patient care may positively influence preparedness in those level 1 milestones. Additionally, several of these 11 events contained hands-on skills training, so this finding could also indicate that students show better response to an active learning setting, allowing them to feel more confident in these categories. 

Lamba et al. previously studied the effects on self-reported confidence after hosting a procedures workshop on intubation, thoracostomy, and central venous catheterization, which fall under the competency of patient care ( 8). The previous study was similar to ours in having a small sample size surveyed before and after an intervention workshop; however, the study was performed on EM-bound senior medical students rather than first- or second-year students. Our study showed similar results in that there was a significant increase in self-reported preparedness for patient care after the elective.

Practice-based performance improvement is a theme seen throughout events in the elective as this study took place in an academic institution with several areas of ongoing research and emphasis on research-based improvement practices. This environment may have played a role in increasing preparedness in this competency. 

System-based practice also showed significant increases in perceived preparedness. The level 1 milestone for this sub-competency SBP2 is that a student is able to describe members of the ED team and their roles (e.g., nurses, techs, security). This skill is best taught through interactions with attendings or students in their third or fourth years and through shadowing. Though skills acquired through shadowing were left open for students to select which ones they would receive credit for, the other events that covered SBP2 involved direct interactions with attendings and/or fourth-year medical students. 

The remaining competencies of professionalism and interpersonal and communication skills (PROF1, ICS1, ICS2) require consistent training and are not readily addressed in the types of events offered through the elective that are more geared towards procedural skills and talk shops. The post-match panel was notable for being moved to a virtual setting this year due to COVID-19 pandemic restrictions, which may have resulted in less engagement with the panelists than has been observed in similar events in previous years. Overall, this may point to the notion that professionalism and interpersonal communications skills are not easily taught or improved upon by a short elective course. In a survey conducted by Stehman et al. regarding assessment of the competency of professionalism, non-technical skills were most commonly assessed by faculty evaluation and only 11.2% of the survey respondents felt that this method of assessment of professionalism was very effective ( 9, 10). In our study, there was no significant increase in perceived preparedness in the non-technical competencies; taken together with the previous study, our results suggest that it may be difficult to objectively assess these skills.

There was no significant increase in interest in EM after the curriculum, though the baseline median level of interest was already the highest of all the survey categories at “very interested.” Students who were already interested in EM may have been more likely to take the elective initially, and those whose interest was consistent throughout the year may have been more likely to complete the elective, resulting in the insignificant change at the end. Students who lost interest in emergency medicine might have dropped the elective and, therefore, potentially biased the sample by not completing research surveys.

### Limitations

One of the limitations of our study is its sample size. Although 22 students completed the elective, we only received 13 complete pre- and post-curriculum sets of data. It is likely that the respondents who were lost to follow-up were those who did not complete the elective. Within the 13 complete data sets, it is also impossible to track which event each of the respondents attended due to the de-identified format of the survey. This absence of tracking is important because it is possible to achieve all the milestones through a number of combinations of events, so the learning environments for each of the 13 students may not have been identical. 

Additionally, there may be volunteer bias due to our use of convenience sampling. The students participating in the surveys were those already enrolled in the elective at the beginning of the academic year and completed it by the end of the year. The students who completed the elective may have been more likely to report increased preparedness due to higher interest in EM-related topics at baseline. Another limitation of our study was the fact that our surveys were not previously validated. Due to the novel design and focus of our study, there were no previously validated surveys that addressed the main hypothesis of this study. 

In our study design, students self-reported their perceived level of preparedness in achieving level 1 milestones, rather than having a standardized method of measuring preparedness in the form of a post-curriculum assessment. For addressing this limitation, future elective courses could have third- or fourth-year medical students or EM residents evaluate the first- and second-year students before and after the elective in a practical skills session or through written exam. Quinn et al. report a method of evaluating students rotating through EM based on milestone achievement through the use of faculty evaluations and quizzes ( 9). Future studies using our elective could develop a more formal assessment of skills in a similar way. 

Another limitation was accommodating for COVID-19 social distancing restrictions and implementation of remote learning so that our final event (Post-Match Panel) was held over video conferencing and may have limited the interaction that normally would be facilitated in a live event. Lastly, it is important to acknowledge the possibility of confounding, as this elective ran concurrently with the MS1 and MS2 medical school curricula. It is possible that increases in perceived preparedness observed in the survey data could be influenced by reinforcement of material in mandatory courses. 

## Conclusion

Implementing a milestones-based curriculum during the pre-clinical years may better prepare the students interested in pursuing EM for meeting level 1 milestones prior to residency stage. This elective can be readily recreated in other programs by creating events that broadly encompass many aspects of EM. Examples include case studies, skills workshops, and interactions with attendings (e.g., lunch/dinner talks). To capture more abstract skills such as professionalism and interpersonal communication, events targeting these skills should be offered. Further investigation in the form of pre- and post-curriculum testing specific to each emergency medicine milestone is warranted to validate these results and better assess the true extent of achieving level 1 milestones. 
